# Host-derived pathogenicity islands in poxviruses

**DOI:** 10.1186/1743-422X-2-30

**Published:** 2005-04-11

**Authors:** Melissa Da Silva, Chris Upton

**Affiliations:** 1 Department of Biochemistry and Microbiology, University of Victoria, Victoria, Canada

## Abstract

**Background:**

Poxviruses are important both as pathogens and as vaccine vectors. Poxvirus genomes (150–350 kb) consist of a single linear dsDNA molecule; the two polynucleotide strands are joined by short hairpin loops. The genomes encode highly conserved proteins required for DNA replication and mRNA transcription as well as a variable set of virulence factors; transcription takes place within the cytoplasm of the host cell. We are interested in evolution of poxvirus genomes and especially how these viruses acquire host-derived genes that are believed to function as virulence factors.

**Results:**

Using a variety of bioinformatics tools, we have identified regions in poxvirus genomes that have unusual nucleotide composition (higher or lower than average A+T content) compared to the genome as a whole; such regions may be several kilobases in length and contain a number of genes. Regions with unusual nucleotide composition may represent genes that have been recently acquired from the host genome. The study of these genomic regions with unusual nucleotide content will help elucidate evolutionary processes in poxviruses.

**Conclusion:**

We have found that dotplots of complete poxvirus genomes can be used to locate regions on the genome that differ significantly in A+T content to the genome as a whole. The genes in these regions may have been acquired relatively recently from the host genome or from another AT-rich poxvirus.

## Background

Poxviruses comprise a family of large dsDNA viruses that replicate in the cytoplasm of eukaryotic cells; they have a wide host range that includes insects, reptiles, birds and mammals [1]. The poxviruses are relatively self-sufficient and encode proteins responsible for the virion structure, DNA replication, mRNA transcription as well as a number of virulence factors [1]. Most poxviruses are moderately AT-rich (55–75%), but there are several exceptions. The known insect poxviruses are extremely AT-rich (82% A+T) whereas molluscum contagiosum virus (MOCV-1) and the known parapoxviruses are AT-poor (36–37% A+T) [1].

**Figure 1:**
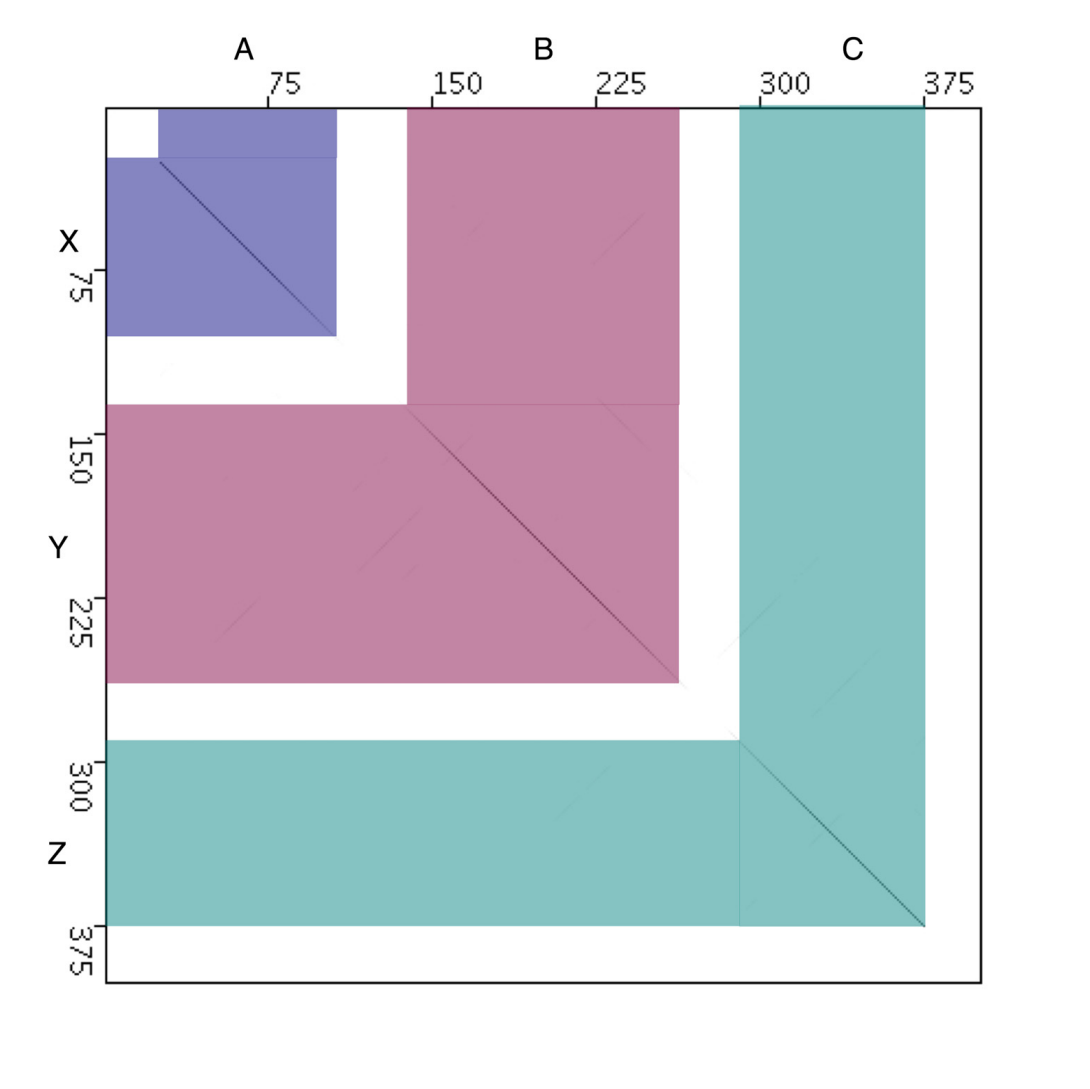
An example of a typical dotplot. Regions on the x-axis that are identical or similar to regions on the y-axis are marked A, B and C and correspond to regions X, Y, and Z on the y-axis respectively. Regions that correspond to each other are also colour-coded in blue for regions A and X, pink for regions B and Y and turquoise for regions C and Z.

When making comparisons of large viral genomes such as poxviruses there are several approaches depending on the level of resolution that is required. Although dotplots can be used for any length of DNA sequence and also for protein sequences, they are especially useful when trying to get a global view of the relationship between large DNA sequences. Since they generate a graphical view of sequence similarity, some relationships between the sequences may be apparent that would otherwise be missed in text formatted alignments [2]. In the simplest form of a dotplot, one sequence is placed along the x-axis and the other sequence along the y-axis to create a matrix (Figure 1). Wherever one nucleotide of one sequence is identical to a nucleotide of the other sequence, the appropriate cell of the matrix is filled. Most dotplot programs, however, use a sliding window of user-defined size, and a measurement of similarity between the two windows instead of comparing each nucleotide along the genomes. When plotting the data, a greyscale can be used to record differing degrees of similarity. If the two sequences are identical, a fully black diagonal line is observed on the dotplot comprised of many dots, each drawn for a window that matches an identical window on the second sequence. The background of the dotplot is therefore comprised of greyscale dots representing random matches of varying similarity between the windows used to scan the two sequences. Figure 1 shows a simplified example of a typical dotplot. For each region of the sequence plotted on the x-axis that is identical to the sequence on the y-axis, a diagonal line is plotted and, for the sake of this example, shaded an appropriate colour to show where each region is identical. Dotter [2] and our java version of this program (JDotter) [3] are especially useful implementations because the window size and degree of similarity used for the dotplot display can both be manipulated on the fly, without recalculation of the whole plot which is CPU intensive. Changing these parameters can help visualize regions of relatively low similarity or to enhance/decrease the background matches in the plot.

**Figure 2:**
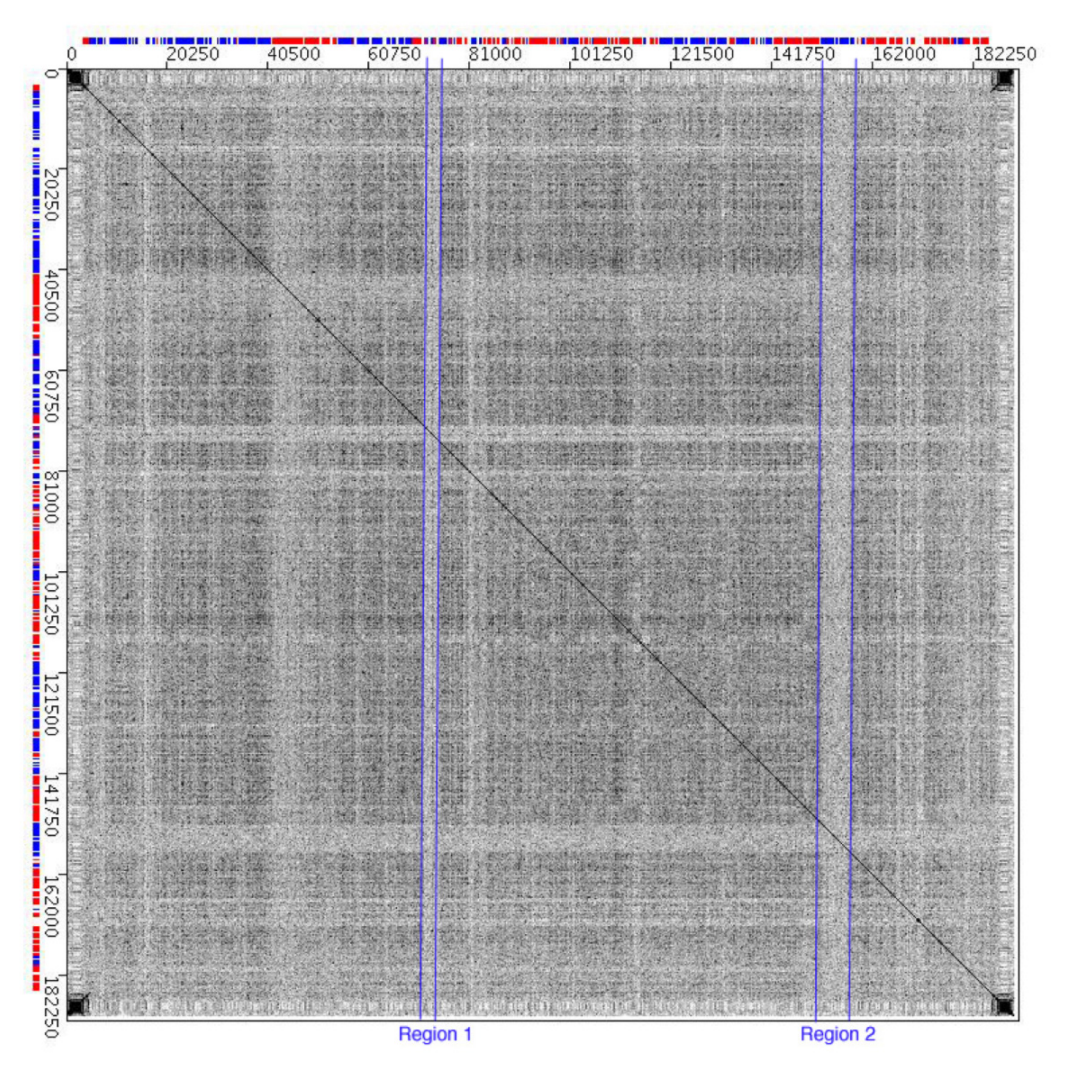
Dotplot depicting a comparison of molluscum contagiosum virus genome to itself. Region 1 begins at position 71,550 bp and ends at position 74,790 bp and contains 5 genes. Region 2 begins at position 151,470 bp and ends at 157,950 bp and contains 5 genes. Blue and red bars across each axis represent each gene as it is located on the MOCV-1 genome with red bars representing genes located on the top strand and blue bars representing genes located on the bottom strand.

When viewing many of the dotplots comparing different poxvirus genomes plotted against each other and themselves, we observed unexpected striped patterns in the background of the dotplots (Figure 2). These unusual non-random banding patterns suggested that the composition of discrete regions of the genomes differed significantly from the overall composition of the poxvirus genomes.

## Results and discussion

**Figure 3:**
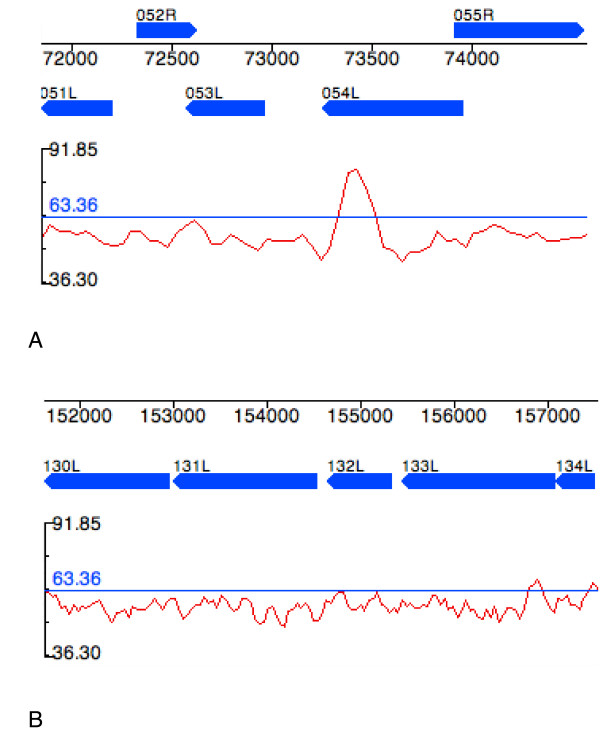
G+C composition plots created using viral genome organizer (VGO) [14] using a window size of 135 bp for (A) region 1 of Figure 2 and (B) region 2 of Figure 2. Scale on left hand side of plot shows the maximum (91.85%) and minimum (36.30%) G+C content of the MOCV-1 genome. Line drawn through the plot indicates the average G+C content of the MOCV-1 genome (63.36%). Numbered blue bars represent the genes in each region and the scale across the top of each panel shows the location of each gene on the genome.

A self-dotplot of MOCV-1, where the viral genome is plotted on both the x- and y-axes, is shown in Figure 2. Since the comparison uses the identical genome on each axis, there is an unbroken diagonal line running from the upper left corner to the lower right corner of the dotplot. A number of horizontal and vertical stripes can be seen scattered throughout the plot; such patterns are observed in most poxvirus genome self-plots although the intensity varies considerably between stripes in a single plot and are usually more intense in those genomes with the most extreme nucleotide composition, A+T or G+C rich genomes. Two of the most striking regions for MOCV-1 are marked on Figure 2. Regions 1 and 2 are located at 71,550 – 74,790 bp and 151,470 – 157,950 bp on the MOCV-1 genome, respectively; each region encompasses 5 genes. It was evident from the dotplot itself that sets of small DNA repeats, which would appear as short parallel lines, were not responsible for generating these unusual stripes in the background of the plot. Therefore, the hypothesis that variations in nucleotide composition were responsible for the patterns was tested. Figure 3 shows that these two regions marked in Figure 2 do indeed have a G+C composition significantly lower than the genome average. Regions 1 and 2 have G+C averages of 52.58% and 50.38% respectively, whereas the G+C composition of MOCV-1 is 63.36% (also shown as a horizontal line in Figures 3A and 3B).

The genes present in regions 1 and 2 are listed in Table 1 together with the A+T content, G+C content and putative function. Region 1 contains 5 genes (MOCV-1-051L, MOCV-1-052R, MOCV-1-053L, MOCV-1-054L, MOCV-1-055R), two of which have unknown function (MOCV-1-052R and MOCV-1-055R). Interestingly, three of the five genes in region 1 show significant similarity to each other. The gene product of MOCV-1-054L is a secreted glycoprotein that binds interleukin-18 [4] and shows some sequence similarity to human and mouse IL-18 binding proteins with three conserved cysteine residues shared between the human and MOCV-1 IL-18 binding proteins [5]; although no function has been predicted for the hypothetical products of related genes MOCV-1-051L and MOCV-1-053L, they are predicted to be secreted from infected cells. Since the other poxviruses that encode an ortholog of the MOCV-1 interleukin-18 binding protein do not contain orthologs of MOCV-1-051L and MOCV-1-053L, it appears that these genes may have arisen from duplications of MOCV-1-054L after the divergence of MOCV from the other poxvirus families.

**Table 1:** Description of genes in regions 1 and 2. Gene names, protein length, AT%, GC% and putative function of all genes described located in regions 1 and 2 (Figure 2).

Region	Gene Name	Protein length (amino acids)	G+C%	A+T%	Putative Function
1	MOCV-1-051L	120	55.92	44.08	Secreted glycoprotein
	MOCV-1-052R	100	54.54	45.54	Unknown
	MOCV-1-053L	133	55.47	44.53	Secreted glycoprotein
	MOCV-1-054L	235	58.05	41.95	Secreted IL-18 binding protein
	MOCV-1-055R	216	55.76	44.24	Unknown

2	MOCV-1-130L	451	57.01	42.99	A-Type inclusion (ATI) protein
	MOCV-1-131L	513	56.48	43.52	Intracellular mature virion surface protein (ATI-factor)
	MOCV-1-132L	229	58.7	41.31	Unknown
	MOCV-1-133L	546	57.4	42.6	Intracellular mature virion surface protein (ATI-factor)
	MOCV-1-134L	141	58.22	41.78	Intracellular mature virion membrane protein (associated with virus entry)

Region 2 is also comprised of 5 genes (MOCV-1-130L, MOCV-1-131L, MOCV-1-132L, MOCV-1-133L, and MOCV-1-134L). Three of the five genes (MOCV-1-130L, MOCV-1-131L and MOCV-1-133L) have low, but significant, similarity to each other and also to both the A-type inclusion (ATI) proteins and orthologs of the vaccinia virus P4c gene (A26L; strain Copenhagen). In some strains of cowpox virus, the ATI protein functions to surround intracellular mature virus (IMV) particles in the cytoplasm of the host cell with the P4c protein playing a role in directing the IMV particles to the A-type inclusions [6,7]. Some orthopoxviruses such as vaccinia and variola encode a presumed non-functional, truncated ATI protein that is incapable of forming occluded ATIs suggesting that ATI formation is not essential for virus survival in the host cell [8]. Little is known about the role of the ATI proteins in the MOCV-1 replication cycle, however, given that these proteins are truncated compared to their cowpox orthologs, it is likely that they have a somewhat different, if any, function in MOCV-1. There are two other genes in region 2; MOCV-1-132L is not found in any other poxvirus and its function is unknown, and MOCV-1-134L is conserved in all poxviruses and shows significant sequence similarity (50% amino acid identity) to the A28L gene of vaccinia virus strain Copenhagen and is an intracellular mature virion membrane protein which is associated with virus entry into the host cell, [9,10].

There are several possible explanations for the observed differences in G+C content of the genes in regions 1 and 2 compared to the rest of the genome. The MOCV-1-054L gene in region 1, for example, was most likely recently acquired from an AT-rich host or from an AT-rich virus with subsequent duplications and divergence resulting in the MOCV-1-051L and MOCV-1-053L genes. Since these genes make up a relatively AT-rich region on the MOCV-1 genome, they may have served as a target site for the acquisition, presumably through non-homologous recombination, of genes MOCV-1-052R and MOCV-1-055R neither of which have been assigned a function. Given that the ATI genes found in region 2 are relatively AT-rich compared to the MOCV-1 genome, these genes may have also been acquired relatively recently from another AT-rich poxvirus. Similar to the genes in region 1, an initial acquisition event leading to an AT-rich region may have led to further acquisition events and the creation of region 2. Although the MOCV-1-134L gene appears to have been acquired from an AT-rich poxvirus, the situation is not simple because the gene has been found to be essential for vaccinia virus entry into the host cell [9,10]. One explanation is that an essential MOCV-1 ortholog was replaced by a similarly functioning gene from an AT-rich poxvirus, thus explaining why it differs in G+C content despite being an essential gene.

It is interesting to note that there is a short sequence in the AT-rich region 1 that has a significantly higher G+C composition than the rest of the region (Figure 3A). This area can be seen on the dotplot as a thin, dark stripe located in the middle of the band for region 1 (Figure 2). This short spike of G+C rich DNA is located at the 3' end of the MOCV-1-054L gene in a region that does not align with either of the two related MOCV-1 genes (MOCV-1-051L and MOCV-1-053L) indicating that this region may have also been acquired relatively recently; this region results in an in-frame insertion in the predicted MOCV-1-054L polypeptide.

**Figure 4:**
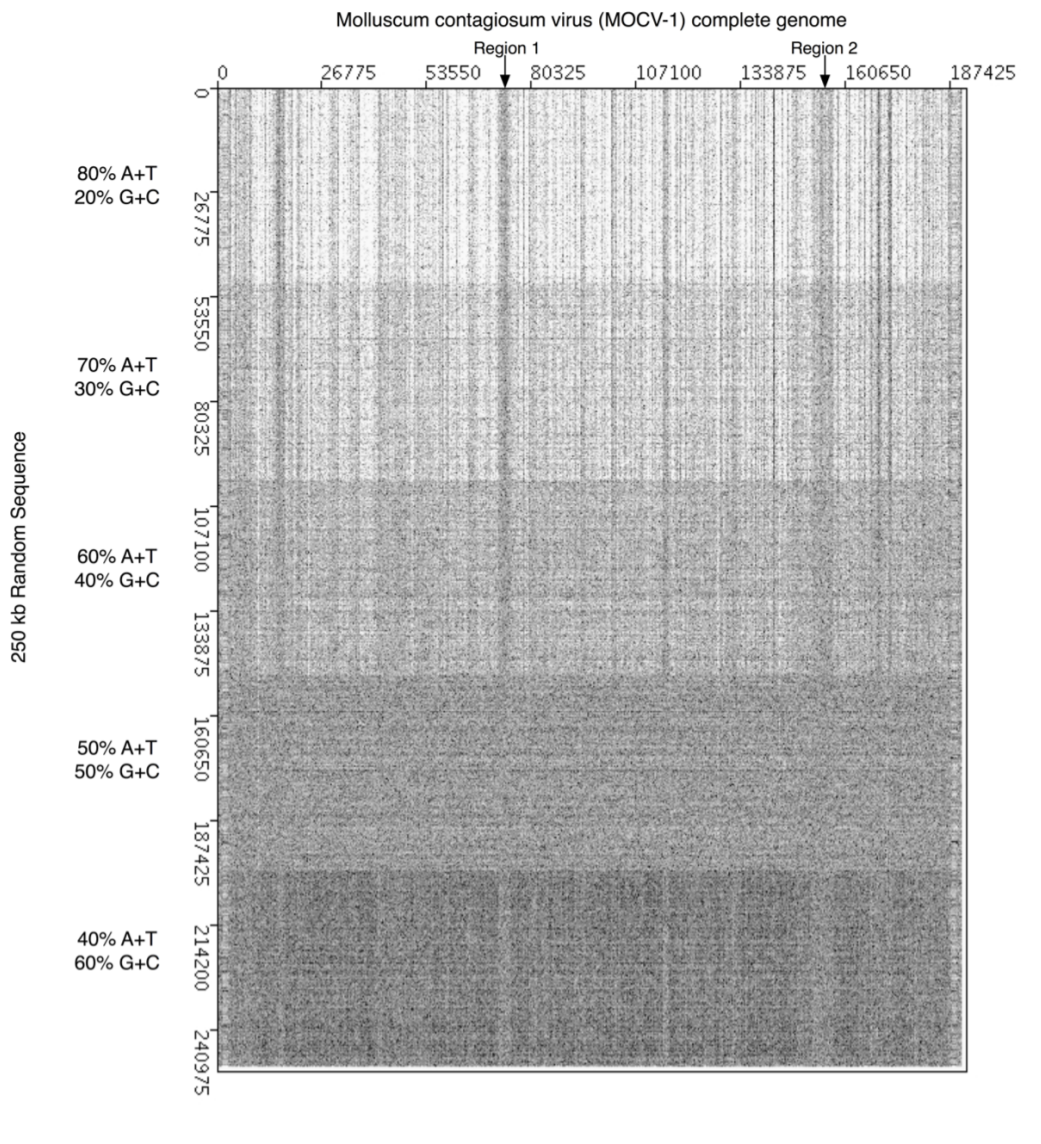
Dotplot comparing molluscum contagiosum virus to a randomly generated 250 kb sequence made up of 50 kb segments with differing A+T content which is indicated on the dotplot. Regions corresponding to the regions seen in Figure 2 are indicated with arrows.

To confirm that the unusual stripes seen in the background of the MOCV-1 dotplot could be due to a lower than average G+C content of these particular regions of the genome, a dotplot was created with the MOCV-1 genome plotted on the x-axis against a 250 kb DNA sequence consisting of five 50 kb randomized DNA segments with specific G+C content on the y-axis (Figure 4). Regions 1 and 2 are marked on the figure and the individual 50 kb segments are clearly visible on the dotplot. As the G+C content increases along the 250 kb sequence on the y-axis, the stripes seen for regions 1 and 2 change colour from dark to light; this reflects a reduction in random matches between the two sequences. At 60% G+C content, the bands for both regions 1 and 2 appear similar to those observed in the MOCV-1 self-dotplot (Figure 2). When the randomized sequence has a G+C content of 50%, the stripes for regions 1 and 2 appear to disappear, as they merge with the background, indicating that the G+C content of these regions is approximately 50%. As the G+C content further decreases to less than 40%, the bands seen for regions 1 and 2 are again visible but are the negative image of those seen in Figure 2; the bands are darker than the background because these regions have a G+C content that is more similar to the randomized sequence than the rest of the MOCV-1 genome. Similarly, each of the 50 kb segments of the random sequence used for Figure 4 produce a different level of background matches in the dotplot and appears as a broad horizontal stripe.

Locating regions of differing G+C content using dotplots can be a difficult task given that the intensity of the banding patterns observed are often diminished in poxvirus genomes that are not extremely AT- or GC-rich. However, we noticed that these banding patterns were more distinct when comparing the MOCV-1 genome to a random sequence with increasing A+T content (Figure 4). For example, areas on the MOCV-1 genome with higher than average G+C content that were previously undetected when observing a self-plot of the MOCV-1 genome can be visualized on the dotplot of the MOCV-1 genome versus a random sequence with decreasing A+T content (compare Figures 2 and 4). Thus this type of comparison can be used to enhance recognition of regions of unusual nucleotide composition.

In order to further illustrate the differences between the genes in regions 1 and 2 compared to the whole genome, the codon usage of 49 MOCV-1 genes that are conserved in all poxviruses was compared to the codon usage of the 10 genes in regions 1 and 2. The mean relative synonymous codon usage (RSCU) values of the two datasets were compared using a Student's T-test. The codons that were found to have statistically different codon usage between the two datasets are listed in Table 2. We found that the codon usage of 43 of 62 codons (69%) was statistically different between all genes in regions 1 and 2 and the 49 conserved MOCV-1 genes. Codon usage was deemed statistically different when the p-value was less than 0.05. The amino acids that have at least 1 codon with statistically equal RSCU values are leucine, isoleucine, valine, serine, proline, threonine, alanine, arginine, glycine and two stop codons (UAA and UAG).

**Table 2:** Codon usage differences between regions 1 and 2 and 49 MOCV-1 genes conserved in all poxviruses. The codon usage between regions 1 and 2 was first determined to be statistically equal and was then used to compare to the codon usage of 49 MOCV-1 genes that are conserved in all poxviruses. Student's t-test was used to compare codon usage with null hypothesis assuming codon usage was equal.

Amino Acid	Fraction of codons with different usage	Codons that have statistically different usage*
Phe	2/2	UUU, UUC
Tyr	2/2	UAU, UAC
His	2/2	CAU, CAC
Gln	2/2	CAA, CAG
Asn	2/2	AAU, AAC
Lys	2/2	AAA, AAG
Asp	2/2	GAU, GAC
Glu	2/2	GAA, GAG
Cys	2/2	UGU, UGC
Leu	4/6	UUA, UUG, CUU, CUG
Ile	1/3	AUC
Val	2/4	GUU, GUG
Ser	2/6	UCU, AGC
Pro	3/4	CCU, CCC, CCA
Thr	2/4	ACU, ACA
Ala	2/4	GCU, GCA
Arg	5/6	CGU, CGC, CGA, CGG, AGA
Gly	3/4	GGU, GGC, GGA
Stop	1/3	UGA

*Codon usage was considered different if p-value < 0.05

In order to determine whether the genes in regions 1 and 2 were recently acquired from the host genome, the codon usage of the genes in regions 1 and 2 was compared to the codon usage of 50 human genes (MOCV-1's natural host), using the same method used for the comparison of regions 1 and 2 with the 49 conserved MOCV-1 genes. The codon usage of the genes in regions 1 and 2 was found to be 68% (48/62 codons) similar to the codon usage of the 50 human genes tested (Table 3). As a control, the codon usage of the same 50 human genes was compared to the codon usage of the 49 conserved MOCV-1 genes and was found to be statistically different in 56 of 62 codons (90%) (Table 4).

**Table 3:** Codon usage differences between regions 1 and 2 and 50 human genes.

Amino Acid	Fraction of codons with different usage	Codons that have statistically different usage*
Phe	0/2	-
Tyr	0/2	-
His	1/2	CAC
Gln	0/2	-
Asn	0/2	-
Lys	0/2	-
Asp	2/2	GAU, GAC
Glu	2/2	GAA, GAG
Cys	0/2	-
Leu	2/6	UUG, CUG
Ile	0/3	-
Val	0/4	-
Ser	1/6	UCG
Pro	2/4	CCU, CCG
Thr	2/4	ACC, ACG
Ala	2/4	GCU, GCG
Arg	4/6	CGU, CGC, AGA, AGG
Gly	0/4	-
Stop	2/3	UAG, UGA

*Codon usage was considered different if p-value < 0.05

**Table 4:** Codon usage differences between 49 MOCV-1 genes that are conserved in all poxviruses and 50 human genes.

Amino Acid	Fraction of codons with different usage	Codons that have statistically different usage*
Phe	2/2	UUU, UUC
Tyr	2/2	UAU, UAC
His	2/2	CAU, CAC
Gln	2/2	CAA, CAG
Asn	2/2	AAU, AAC
Lys	2/2	AAA, AAG
Asp	2/2	GAU, GAC
Glu	2/2	GAA, GAG
Cys	2/2	UGU, GUC
Leu	6/6	UUA, UUG, CUU, CUC, CUA, CUG
Ile	3/3	AUU, AUC, AUA
Val	3/4	GUU, CUA, GUG
Ser	5/6	USU, UCA, UCG, AGU, AGC
Pro	4/4	CCU, CCC, CCA, CCG
Thr	3/4	ACU, ACA, ACG
Ala	3/4	GCU, CGA, GCG
Arg	6/6	CGU, CGC, CGA, CGG, AGA, AGG
Gly	3/4	GGU, GGC, GGA
Stop	2/3	UAG, UGA

*Codon usage was considered different if p-value < 0.05

Since the codon usage of the genes in regions 1 and 2 was found to be statistically different to the codon usage of 49 conserved MOCV-1 genes for 69% of the codons tested yet was found to be only 34% different from the codon usage of the 50 human genes tested, it is reasonable to assume that these genes may be recent acquisitions from the host genome or a poxvirus genome with higher A+T composition. Interestingly, since the initial submission of this manuscript, a paper has been published which identifies the MOCV-1 interleukin-18 binding protein (as well as several other poxvirus proteins) as a possible horizontally transferred gene [11], which further supports our hypothesis that the genes in regions 1 and 2 were likely acquired from external sources.

Pathogenicity islands (PAI) in bacterial genomes contain several distinct structural features, which include the presence of virulence genes within the PAI, a nucleotide composition of the PAI that is different from the remainder of the genome, and occupancy of large regions of the bacterial chromosome [12]. The genes in regions 1 and 2 also occupy a relatively large region of the MOCV-1 genome, and some of the genes in these regions are known virulence factors with the function of the remaining genes yet to be determined. Thus, we suggest that these regions may be analogous to bacterial pathogenicity islands. This notion is further supported by the observations in this manuscript, which showed the differences in nucleotide composition and codon usage of the genes in regions 1 and 2.

## Conclusion

The data presented in this paper suggest that the unusual striped banding pattern seen on the dotplots of poxvirus genomes is due to differences in nucleotide content of the genes in these regions compared to the remainder of the genome. The difference between the genes in these regions and 49 MOCV-1 genes that are conserved in all poxviruses was further shown by comparing the codon usage of 62 codons in each of these datasets. The codon usage was found to differ significantly in 69% of the codons tested. The codon usage of the genes in regions 1 and 2 was found to be statistically similar to 50 human genes in 68% of the codons tested.

We conclude that the genes in regions 1 and 2 have a relatively low G+C content and that they may have been acquired from either an AT-rich host or virus making these regions possible novel pathogenicity islands in the MOCV-1 genome. A future survey of all other poxviruses is needed in order to determine the extent of these possible gene acquisitions.

## Methods

### Creation of dotplots

Dotplots for the molluscum contagiosum virus genome were created and visualized using JDotter with a default window size of 26 nucleotides using a minimum cut-off score of 40 and a maximum cut-off score of 100 on the GreyMap tool in order to better visualize the unique background patterns [3].

The dotplot shown in Figure 4 was created using the Dotter program with the complete molluscum contagiosum virus genome plotted on the x-axis and a 250 kb random sequence with increasing G+C content plotted on the y-axis [2]. The 250 kb sequence was created using DNACreator a program that creates a random sequence with a given G+C content [13]. Segments of 50 kb and varying G+C content were created and concatenated into one 250 kb sequence that contained an increasing G+C content.

### G+C composition plots

The G+C composition of regions 1 and 2 was plotted using the "nucleotide base content" feature of the program Viral Genome Organizer (VGO) [14]. This feature calculates and plots the G+C content of the entire genome using a user-specified window size.

### Codon usage

#### Comparison between genes in regions 1 and 2 and 49 conserved MOCV-1 genes

The program CodonW was used to calculate the Relative Synonymous Codon Usage (RSCU) values of 49 molluscum contagiosum virus genes that are conserved in all poxviruses, and the 5 genes comprising each of region 1 and 2 (Figure 2 and Table 1) [15]. The mean RSCU values for each codon excluding the codons for methionine and tryptophan (62 in total) from regions 1 and 2 were initially compared to each other using a two-tailed Student's T-test with the null hypothesis that the means are equal being rejected only if the p-value was less than 0.05. From the results of these T-tests, the RSCU values for each region were pooled in order to create a larger data set to perform subsequent Student's T-tests. The mean RSCU values for four codons in regions 1 and 2 were found to be statistically different and were therefore treated as individual data sets when subsequent T-tests were performed. These codons were CCA (proline), CAU and CAC (histidine), and CAG (glutamine).

With the RSCU values from regions 1 and 2 being pooled for all but 4 codons, the mean values for this pooled dataset were compared to the mean RSCU values obtained for the 49 conserved MOCV-1 genes again using a two-tailed Student's T-test. The null hypothesis that mean RSCU values from the two datasets are equal was rejected only when the p-value was less than 0.05.

#### Comparison between MOCV-1 and 50 human genes

Using the above outlined method, the mean RSCU values for the genes in regions 1 and 2 were compared to the mean RSCU values of 50 randomly selected human genes. The 50 human genes were randomly selected from the dataset used by the codon usage database at the Kazusa DNA Research Institute [16] that consists of 76,893 human coding sequences that were taken from the NCBI Genbank FlatFile release 145.0 (January 25, 2005). The comparison of the codon usage of 50 human genes with the codon usage of 49 MOCV-1 genes was performed using the same methods outlined above. In each of the analyses performed, the null hypothesis that mean RSCU values from the two datasets are equal was rejected when the p-value was less than 0.05

## Competing interests

The author(s) declare that they have no competing interests.

## Authors' contributions

CU initiated the project and edited the manuscript; MDS performed all the work and wrote the manuscript.
